# Short term outcomes of robotic surgery after neoadjuvant treatment for locally advanced NSCLC: a comparison with the open approach

**DOI:** 10.3389/fonc.2025.1579943

**Published:** 2025-08-06

**Authors:** Filippo Tommaso Gallina, Riccardo Tajè, Daniele Forcella, Gabriele Alessandrini, Francesco Vizza, Rachele Bovini, Paolo Visca, Isabella Sperduti, Federico Cappuzzo

**Affiliations:** ^1^ Thoracic Surgery Unit, IRCCS Regina Elena National Cancer Institute, Rome, Italy; ^2^ Tumor Immunology and Immunotherapy Unit, IRCCS Regina Elena National Cancer Institute, Rome, Italy; ^3^ Doctoral School of Microbiology, Immunology, Infectious Diseases and Transplants, MIMIT, University of Rome “Tor Vergata”, Rome, Italy; ^4^ Medicine and Surgery, University of Rome “La Sapienza”, Rome, Italy; ^5^ Medicine and Surgery, University of Perugia, Terni, Italy; ^6^ Department of Pathology, IRCCS Regina Elena National Cancer Institute, Rome, Italy; ^7^ Biostatistics Unit, IRCCS Regina Elena National Cancer Institute, Rome, Italy; ^8^ Medical Oncology 2, IRCCS Regina Elena National Cancer Institute, Rome, Italy

**Keywords:** lung cancer, neoadjuvant, chemo-immunotherapy, robotic surgery, NLR

## Abstract

**Introduction:**

Despite thoracotomy remaining the gold standard in the treatment of locally advanced NSCLC after induction treatment, robotic surgery may improve perioperative outcomes. The object of this study is to compare robotic surgery with thoracotomy for the treatment of NSCLC after neoadjuvant treatment, analyzing primary the postoperative complications and secondary the length of hospital stay, the daily drainage volume and the neutrophils-to-lymphocyte ratio.

**Methods:**

The study was designed as a single center and retrospective analysis. Patients with locally advanced NSCLC underwent neoadjuvant treatment followed by surgery between 01/2017 and 12/2023 were evaluated.

**Results:**

A total of 60 patients were collected. The most frequent clinical stage was IIIa (38,3%). Platinum based chemotherapy was delivered in 56 patients; it was associated with immunotherapy in 28 cases and to radiotherapy in 14 cases. All the patients underwent lobectomy and systematic lymphadenectomy, 25 via robotic surgery. There was no significant demographic difference between the two cohorts except for preoperative radiotherapy; over one-third of patients in the open cohort received radiotherapy, while no patients in the robotic cohort did (p<0.001). The hospital stay was statistically significantly shorter in the robotic group (6 days (4-17) vs 8 (5 - 29); p=0.02). Postoperative complication rates were lower (42,8% vs 20%, p=0.04) and the daily drainage output was significantly lower (p=0.0001). The NLR evaluated in V postoperative days was significantly lower in the robotic group (3.36 ± 1.99 vs 7.27 ± 2.59, p=0.0001).

**Conclusion:**

Despite significant selection bias between cohorts, particularly regarding the use of preoperative radiotherapy which might have influenced the outcomes, robotic surgery appears feasible and yields comparable short-term outcomes for patients with locally advanced NSCLC following neoadjuvant therapy.

## 1 Introduction

Lung cancer represents the first cause of cancer-related deaths worldwide and its incidence continues to escalate. Surgery represents the primary curative-intent treatment for patients with resectable non-small cell lung cancer (NSCLC) (1). Among cases with either a primary tumor ≥ 4cm or ipsilateral nodal involvement, platinum-based (neo)adjuvant chemotherapy was shown to modestly improve overall survival (OS). The marginal benefits in terms of survival offered by neoadjuvant platinum-based chemotherapy have so far questioned the role of neoadjuvant regimens in thoracic surgery.

To date, minimally invasive surgery, including robotic approach has become the new standard of care for the treatment of early stage NSCLC by demonstrating reduced postoperative pain and pain medication use, shorter hospital stays, and fewer complications (2). Moreover, due to its intrinsic ergonomic characteristics, robotic surgery holds the promise to improve dissection and to ameliorate intraoperative outcome even in more demanding procedures. Despite these advantages, safety and feasibility of robotic surgery in locally advanced resectable NSCLC remains unclear.

Neoadjuvant chemo-immunotherapy has completely changed the treatment paradigm in locally advanced resectable NSCLC. This type of treatment has shown significantly better results compared to standard chemotherapy leading to a worldwide escalation in neoadjuvant chemo-immunotherapy appeal (3–5). The rapid diffusion of neoadjuvant chemo-immunotherapy as well as the need of a prompt reinitiation of treatment in perioperative regimens, have triggered the interest toward robotic surgical approach feasibility in these tumors to reduce surgical invasiveness and improve patients’ adherence to the treatment (6). Nevertheless, robotic surgery compared to traditional surgery may also reduce surgical stress and, consequently, systemic inflammation. In the last few years, systemic inflammation scores such as neutrophils-to-lymphocytes ratio (NLR) has been evaluated as predictive factors of long term outcomes in various types of cancers, including NSCLC. Indeed, a high NLR seems to be associated with worse response to immunotherapy and short DFS and OS (7–10). However, the immune response generated by chemo-immunotherapy is often intense, leading to fibrosis and dense adhesions that can complicate subsequent surgery questioning the safety and feasibility of robotic surgery in this setting. As a result, thoracotomy is still the most frequently offered treatment for locally advanced resectable NSCLC after neoadjuvant chemo-immunotherapy (11, 12).

In this study, we aimed to compare the short-term surgical outcomes of robotic-assisted surgery and thoracotomy in patients with locally advanced NSCLC following neoadjuvant treatment. Specifically, we evaluated the feasibility and safety of robotic surgery in terms of postoperative complications, hospital length of stay, daily drainage output, and changes in the NLR.

## 2 Materials and methods

### 2.1 Study design

This study was conducted as a single-center retrospective analysis. Patients diagnosed with locally advanced NSCLC who underwent neoadjuvant therapy followed by surgical resection between January 2017 and December 2023 were included. Data were retrieved from our institutional lobectomy database, supplemented by a review of medical records, operative reports, and outpatient clinic notes to gather perioperative clinical and pathological characteristics as well as postoperative complications.

### 2.2 Patient selection and data collection

The inclusion criteria encompassed patients aged 18 years or older with a diagnosis of resectable locally advanced NSCLC or oligometastatic disease who received neoadjuvant therapy and subsequently underwent either robotic-assisted surgery or conventional thoracotomy. Conversely, patients with unresectable tumors or early-stage NSCLC were excluded. The decision between robotic-assisted surgery and thoracotomy was made based on surgeon expertise, tumor characteristics (e.g., tumor size, location, and extent of hilar/mediastinal invasion), and patient comorbidities. Additionally, the adoption of the robotic platform in our unit began in 2016, progressively becoming the preferred approach as surgical expertise expanded. Consequently, thoracotomy was more commonly performed in the earlier phase of the study period and was generally reserved for cases presenting with extensive local invasion, particularly those involving the central bronchus or pulmonary artery, or when technical limitations were anticipated. With the maturation of the robotic program, the majority of procedures, including complex resections, are now performed robotically.

The collected data included demographic and clinical information such as date of birth, sex, age at diagnosis, smoking history, ASA score, comorbidities, tumor diameter, clinical TNM stage (cTNM), tumor location, details and duration of neoadjuvant therapy, post-treatment clinical staging (ycTNM), preoperative laboratory results, date of surgery, type of procedure, operative time, intraoperative complications and conversions, drainage volume, postoperative complications within 30 days, postoperative blood tests, hospital length of stay, histopathological findings, pathological TNM stage (ypTNM), number of lymph node stations sampled, number of lymph nodes resected, number of metastatic lymph node stations, number of metastatic lymph nodes, and pathological response to therapy. The intraoperative parameters analyzed included operative time, estimated blood loss, type of procedure performed, number of lymph node stations sampled, and intraoperative complications.

Neoadjuvant treatment strategies were determined by a multidisciplinary tumor board according to the availability of the treatment at the time of decision. The majority of patients received platinum-based chemotherapy, either alone or in combination with immunotherapy or radiation therapy. Selected patients with oncogenic driver mutations received targeted therapy. The treatment response was assessed using RECIST criteria, and surgical resection was planned for patients demonstrating disease control or partial response.

### 2.3 Neoadjuvant treatment and resectability assessment

Preoperative staging included whole-body computed tomography (CT) and 18F-fluorodeoxyglucose positron emission tomography (FDG-PET). Bone scintigraphy was performed when clinically indicated. Patients with suspected hilar or mediastinal lymph node metastases underwent non-invasive endoscopic ultrasound-guided biopsy. Resectability was determined before initiating treatment and discussed within a multidisciplinary tumor board involving thoracic surgeons, radiologists, oncologists, radiation oncologists, and pulmonologists.

### 2.4 Preoperative functional assessment

Pulmonary and cardiopulmonary function was assessed through spirometry, diffusion capacity of the lung for carbon monoxide (DLCO), and arterial blood gas analysis. Predicted postoperative (ppo) forced expiratory volume in one second (FEV1), forced vital capacity (FVC), and DLCO values were calculated based on the planned surgical procedure. Additional functional tests, including the six-minute walk test and cardiac stress tests, were conducted in patients with impaired cardiopulmonary function. Performance status was evaluated using the Eastern Cooperative Oncology Group (ECOG) scale. Systemic inflammatory markers, such as NLR, and absolute neutrophil, lymphocyte, and platelet counts, were obtained from blood tests performed within 30 days before surgery. Before surgery, all patients provided informed consent for lobectomy. Those undergoing robotic-assisted thoracic surgery (RATS) were counseled regarding the possibility of conversion to thoracotomy in case of intraoperative technical challenges.

### 2.5 Postoperative management

Postoperative care followed institutional protocols, including routine blood tests and chest X-rays on postoperative days one and five. Neutrophil, lymphocyte, as well as NLR, were recorded from standard postoperative blood tests. Daily chest drainage output and air leaks were documented in clinical records. Chest drains were removed in the absence of air leaks and when daily drainage was below 150 mL. Air leaks persisting beyond five days were classified as prolonged, and patients could be discharged with a Heimlich valve if preferred.

A postoperative outpatient follow-up was conducted one month after surgery by a thoracic surgeon. Standard follow-up included a clinical examination, laboratory tests, and a chest X-ray. Postoperative complications were classified according to standardized guidelines and graded using the Clavien-Dindo classification. Readmission events after discharge were also recorded.

### 2.6 Statistical analysis

Comparisons between groups were performed using the Student’s t-test for continuous variables and either the Pearson chi-square test or Fisher’s exact test for categorical variables. The Fisher’s exact test was applied when at least 20% of contingency table cells had expected counts below five. A p-value < 0.05 was considered statistically significant. The optimal cutoff for NLR was determined via receiver operating characteristic (ROC) curve analysis. Differences in postoperative complications between groups were analyzed using unstratified log-rank tests. Odds ratios (OR) with corresponding 95% confidence intervals (CI) were calculated using univariable and multivariable logistic regression analyses. The variables included in multivariable logistic regression were selected based on established predictive factors and those with a p-value < 0.05 in unstratified log-rank testing. The alpha level for statistical significance was set at 0.05 for all analyses. Data analysis was conducted using IBM SPSS Statistics version 26 (IBM Corporation, Chicago, IL, USA).

## 3 Results

### 3.1 Patients characteristics

A total of 60 patients were included in this retrospective study (**Table 1**). Their median age was 62 (43 - 79), 33 patients were male (55%). Former or active smokers were 52 (86,7%) and the majority of patients had a PS ECOG at diagnosis of 0 and a Charlson comorbidity index of 2 or 3 in 83.3% of patients. The most common clinical stage was IlIa stage, diagnosed in 23 patients (38,3%). Moreover, 11 patients with selected oligometastatic disease that underwent sequential radical metastasectomy followed neoadjuvant treatment and curative resection were also included. Adenocarcinoma was the most common histology.

**Table 1:** Clinical and demographic characteristics.

Variables	Open surgery (nr: 35)	Robotic surgery (nr: 25)	P-value
Gender (Male/Female)	21/13	11/14	0.23
Smoking (nr,%)	28	24	0.19
Age (median, IQR)	64 (51-76)	63 (43-76)	0.93
CC Index (median, IQR)	3 (2-5)	3(2-6)	0.83
FEV1% (median, IQR)	101 (71-121)	103 (69-117)	0.63
FVC% (median, IQR)	100 (69-114)	103 (73-115)	0.10
DLCO% (median, IQR)	81 (69-97)	80 (74-101)	0.28
cSTAGE (IIb-IIIa/IIIb)	20/15	14/11	0.19
Histology			
Adenocarcinoma (nr,%)	29	22	0.2
Solid	7	4	
Micropapillary	3	5	
Mucinous	4	2	
Acinar	15	13	
Squamous cell Carcinoma (n,%)	6	3	0.1
Kind of neoadjuvant			
Platinum-based (nr,%)	34	22	0.27
Immunotherapy (nr,%)	12	17	0.02
Target therapy (nr,%)	0	2	0.1
Radiotherapy (nr,%)	14	0	0.0001
Nr of Cycles (median, IQR)	3 (2-4)	3 (2-5)	0.44
Treatment toxicity (nr,%)	4	5	0.19
Radiological Response (nr,%)	25	22	0.22
pCR (nr,%)	13	15	0.13
Kind of lobectomy			
Standard Lobectomy (n,%)	25	17	0.12
Lobectomy enbloc with lung paranchyma (n,%)	6	4	0.2
Lobectomy with bronchoplasty (n,%)	1	0	0.1
Lobectomy enbloc with aygos	0	1	0.1
Other lobectomies	3	3	0.9
Itraoperative			
Operative time (min)	251 ± 57.97	231 ± 47.91	0.1
Intraoperative blood loss (cc)	350 ± 50	200 ± 25	0.03
Number of resected nodes (median, IQR)	12 (7–18)	12 (8–21)	0.3
Number of harvested nodal station (median, IQR)	5 (4–6)	5 (4–5.5)	0.2
Intraoperative complications (n, %)	3 (8.5)	1 (4)	0.07
Conversion rate (n, %)	–	1 (4)	
Hospital Stay (days, median, IQR)	12.69 ± 11.87	9.08 ± 4.15	0.01
Drainage duration (days, median, IQR)	7.4 ± 6.7	5.3 ± 3.3	0.2

Overall, to 57 patients a platinum based chemotherapy was delivered alone or in combination with immunotherapy (27) and radiotherapy (14). Two patients underwent targeted neoadjuvant treatment with alectinib and one patient immunotherapy alone. A chemotherapy toxicity was experienced by 7 patients. None of the toxicities were associated with immunotherapy or targeted therapy. After systemic treatment, the cases were discussed in the context of a multidisciplinary meeting and the majority of patients showed a partial response (85%).

### 3.2 Comparison robotic vs open

Overall, 35 underwent surgery using the standard thoracotomy (58,3%), while 25 underwent robotic approach (41,7%). The two groups were homogeneous in terms of sex, smoking history, histology, clinical stage. Regarding the kind of neoadjuvant treatment, the chemo-radiotherapy was delivered more frequently in the thoracotomy group (p=0.0001) while chemo-immunotherapy in the robotic group (p=0.02, **Table 1**). The extent of resection was similar between the two groups (**Table 1**).

The mean operative time was similar between the robotic and thoracotomy groups (231.86 ± 47.91 vs. 251.64 ± 57.97 minutes, p=0.1). Estimated intraoperative blood loss was significantly lower in the robotic group (p=0.03). The number of lymph node stations sampled and total lymph nodes resected did not significantly differ between the groups. Intraoperative complications occurred in one case in the robotic group (conversion to thoracotomy due to major bleeding) and in three cases in the thoracotomy group (major bleeding, airway injury, and prolonged hypotension requiring vasopressors).

**Figure 1:**
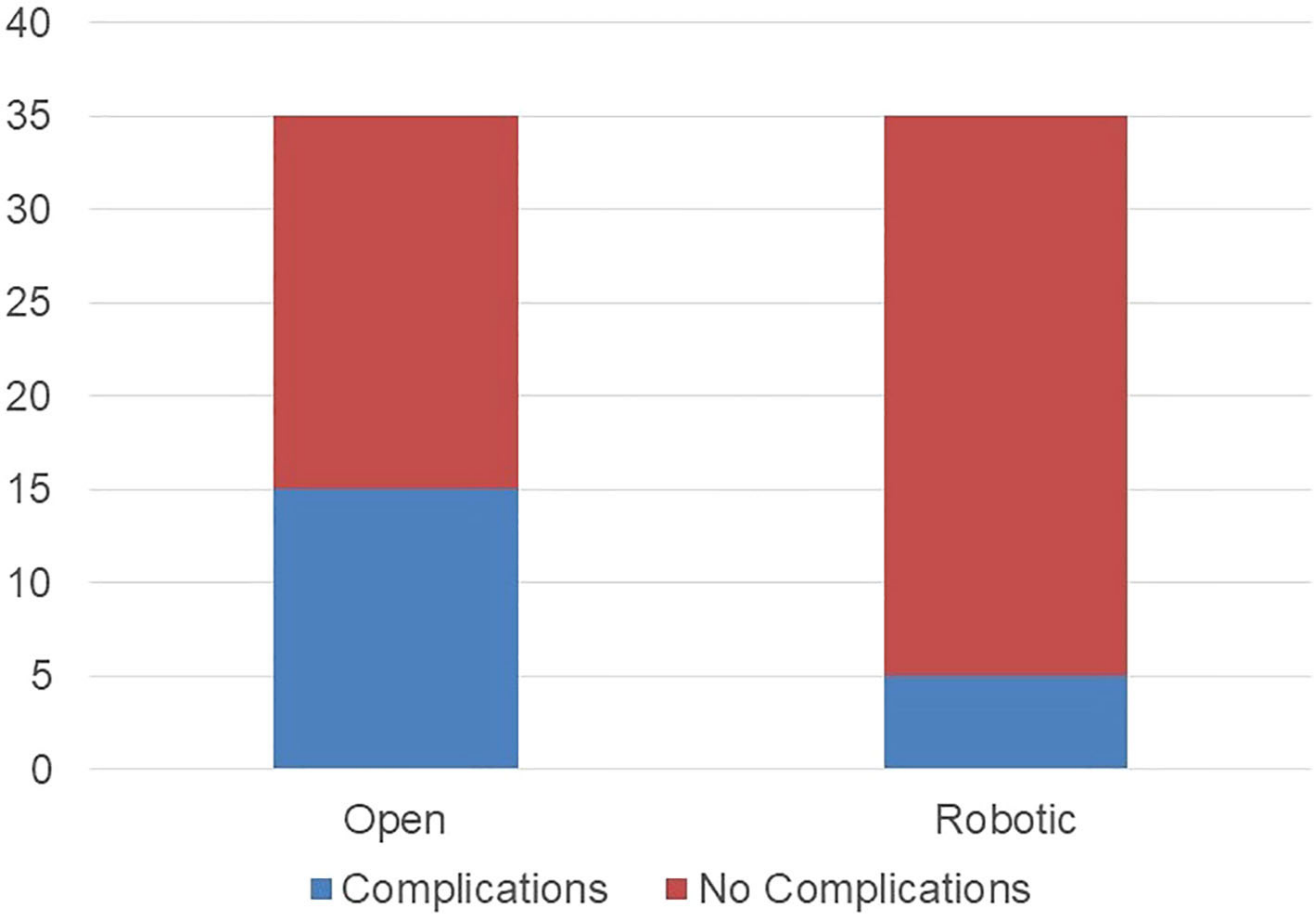
Postoperative complications rate (any grade) in thoracotomy and robotic group.

No differences were observed between groups in terms of any postoperative complications that occurred in a total of 20 patients, 5 in the robotic group and 15 in the thoracotomy group (p=0.1, **Figure 1**). The kind of complications was summarized in **Table 2**. According to the Clavien-Dindo classification, grade 3 complications were significantly more frequent in the thoracotomy group (p=0.04),. One patient in the thoracotomy group died after surgery.

**Table 2:** Postoperative complications and drainage output values.

Variables	Open surgery (nr: 35)	Robotic surgery (nr: 25)
Kind of postoperative complications		
Haemothorax, n (%)	1 (2.8)	0 (0)
Middle Lobe Torsion, n (%)	1 (2.8)	0 (0)
Chronic Respiratory Failure, n (%)	1 (2.8)	0 (0)
Pneumonia, n (%)	2 (5.7)	0 (0)
Empyema, n (%)	1 (2.8)	1 (4)
Atrial Fibrillation, n (%)	2 (5.7)	1 (4)
Prolonged Air Leaks, n (%)	7 (20)	3 (12)
Clavien – Dindo Grading (nr)		
Grade I	2	2
Grade II	8	2
Grade III	3	1
Grade IV		0
Grade V	2	0
Drainage output		
Day 1	380 (255-500)	302 (160-450)
Day 3	301 (190-425)	250 (190-320)
Day 4	290 (50-450)	210 (145-300)
Day 5	220 (150-290)	200 (100-300)

**Figure 2:**
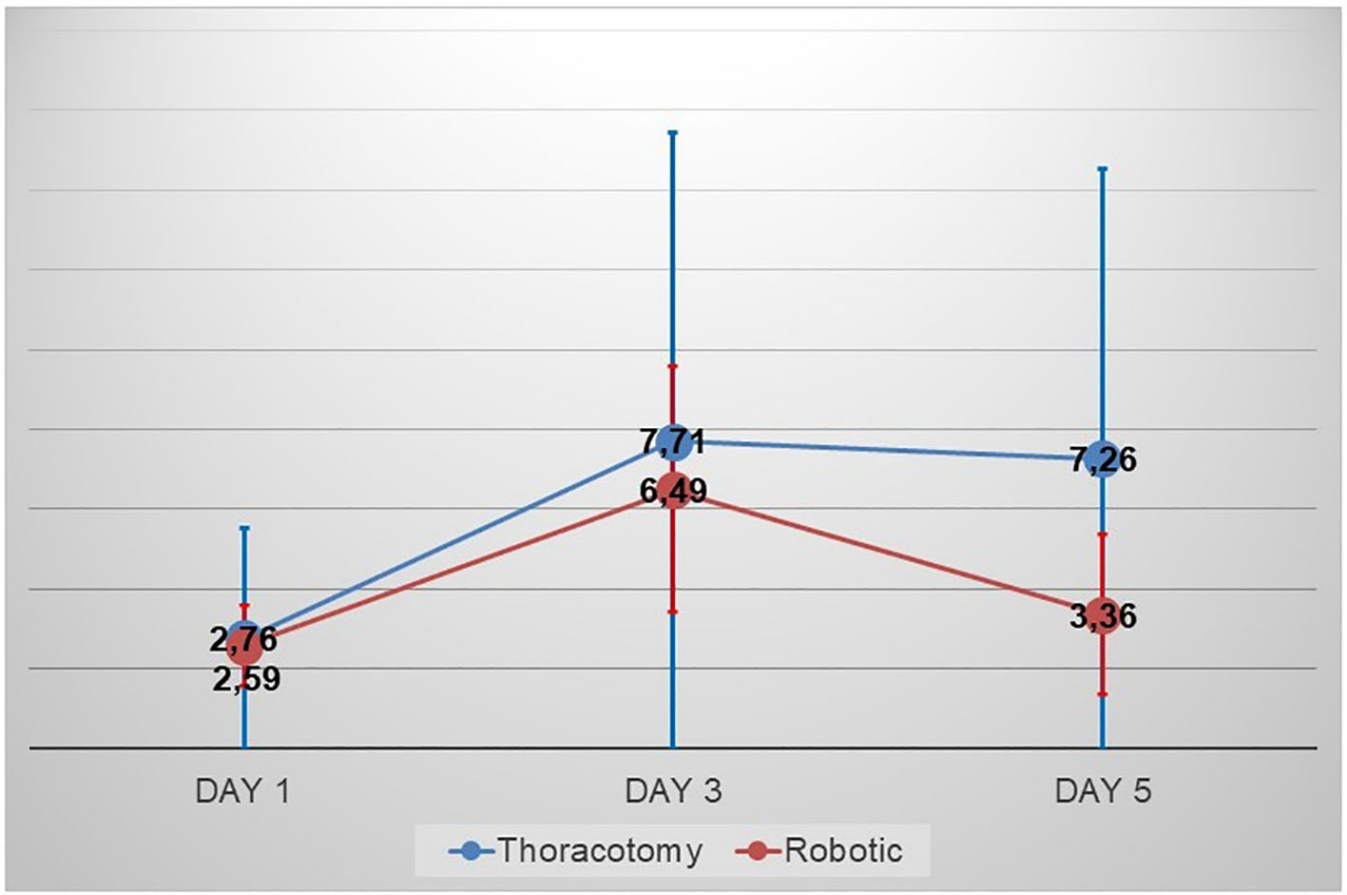
NLR value in preoperative setting, day1 postoperative and day 5postoperative in the thoracotomy and robotic group.

The length of stays, as well as the drainage duration, was numerically shorter in the robotic group 9.08 ± 4.153 vs 12.69 ± 11.87 (p=0.10) and 7.4 ± 6.7 vs 5.3 ± 3.3 (p=0.2). With regards to the daily drainage output (**Figure 2**), the robotic group had a faster decrease of the daily drainage output compared to the thoracotomy group with a median 5 days, 95% CI 3.800 - 6.200 vs 6 days, 95% CI 5.227 - 6.773 respectively (p=0.02). The drainage rate was evaluated at day 1, day 3 and day 5 post op and overall, the robotic group showed a lower rate of drainage output as shown in **Table 2** (p=0.0001). However, the difference between groups in terms of drainage output in day 1 post op was significantly greater (p=0.01) compared to day 5 (p=0.06).

The analysis of neutrophils to lymphocytes ratio changes was shown in the **Table 2**. Despite the comparable value observed in the preoperative (2.76 ± 1.69 vs 2.59 ± 1.01, p=0.67) and in the first postoperative day 7.71 ± 3.88 vs 6.49 ± 3.09, p= 0.185), the analysis of the fifth postoperative day showed a significantly lower value in the 7.26 ± 2.59 in the thoracotomy group vs 3.36 ± 1.99 in the robotic group.

The multivariate analysis of factors associated with postoperative complications showed that open surgery (OR 1.5 95% CI 1.1-2.3, p= 0.03) and a higher value of NLR in the fifth postoperative day (OR 1.7 95%CI 1.3-2.2, p=0.01, **Table 3**).

**Table 3:** Multivariate analysis of factors associated with postoperative complications.

Variables	OR	p-value	95%CI
Univariate			
Open Surgery	1.5	0.03	1.1-2.3
NLR preop	1.1	0.2	0.6-1.7
NLR I postop	0.8	0.4	0.4-2.1
NLR V postop	1.4	0.01	1.3-2.7
Gender	.08	0.3	0.7-1.9
Histology	1.5	0.1	0.5-2.2
Stage	1.4	0.6	0.7-1-9
Smoking History	1.6	0.03	1.1-2.1
CC Index <4	1.5	0.04	1.1-1.6
FEV1% >60%	0.8	0.1	0.3-1.2
Preop chemo-(immunotherapy)	1.1	0.04	1-1.6
Preop radiotherapy	0.9	0.3	0.4-2.2
Number of resected nodes	3.7	0.01	2.2 – 5.9
Multivariate			
NLR V postop	1.7	0.01	1.3-2.2
Smoking History	1.6	0.07	0.8-2.1
CC Index <4	1.8	0.06	0.9-1.6
CC Index <4	1.8	0.06	0.9-1.6
Preop chemo-(immunotherapy)	1.1	0.08	0.7-1.6
Number of resected nodes	2.5	0.02	1.1 – 5.7

## 4 Discussion

In recent decades, the thoracotomy approach has been gradually replaced by minimally invasive techniques for treating early-stage NSCLC (13). These techniques are associated with reduced postoperative pain, shorter immune response times, faster recovery, and better functional and aesthetic outcomes. The advent of robotic surgery brought several technical advantages, such as 3-dimensional visualization, intuitive use of flexible instruments with increased precision, and tremor filtration. Furthermore, the robotic approach demonstrated superior performance in lymph node dissection, which can have significant implications for oncological outcomes (14). The robotic approach is now widely used in early-stage lung cancer, showing improved perioperative outcomes to thoracotomy (15). However, despite these results in early-stage disease, few studies analyzed the outcomes in the post-induction setting.

The findings of our study suggest that robotic surgery offers significant benefits over traditional open thoracotomy in managing locally advanced NSCLC following neoadjuvant treatment. Our results showed that robotic surgery is associated with fewer postoperative complications, shorter hospital stays, lower daily drainage volumes, and a more favorable trajectory of systemic inflammation markers, specifically the NLR that directly impacts the complication rate.

For patients with locally advanced NSCLC following neoadjuvant therapy, the choice of surgical approach remains a matter of debate. While thoracotomy has traditionally been preferred due to concerns regarding adhesions and fibrosis from prior treatment, our study suggests that robotic-assisted surgery can be safely performed with comparable or even superior short-term outcomes. The enhanced visualization and precision offered by the robotic platform may help mitigate challenges related to tissue dissection in post-induction settings.

Several studies focusing on NSCLC have consistently demonstrated a lower incidence of complications associated with robotic surgery compared to open procedures. For instance, Zhang et al. conducted a meta-analysis of 22 studies involving 104,472 patients and reported a reduced rate of complications with RATS over open surgery (16). Our study supports these findings, particularly in the context of locally advanced disease, where the benefits of minimally invasive surgery may be even more pronounced. The faster reduction in daily drainage volume in the RATS group aligns with the findings of Jia Huang et al., who demonstrated that patients treated with robotic approach had significantly shorter drainage tube duration compared to those undergoing open surgery (17). This can be attributed to the precision and reduced tissue trauma associated with robotic surgery, likely leading to quicker resolution of postoperative fluid accumulation.

Inflammation plays a crucial role in perioperative outcomes and tumor progression, as evidenced in recent years. NLR, as a prognostic or predictive marker for individual risk assessment, has been widely used for multiple malignancies. Other inflammatory prognostic markers, such as the PLR and the Glasgow prognostic score (GPS), later modified (mGPS), have shown favorable reports. Many consider NLR a useful, simple, and discriminating independent prognostic marker for survival in various malignancies. The relationship between perioperative systemic inflammation and postoperative complications in several malignancies, such as rectal, gastric, and gynecologic cancers, and some non-cancer diseases, has been documented. For instance, the mGPS was reported to show a significant association with postoperative complications after elective bowel resection in patients with Crohn’s disease and ulcerative colitis. Kenichi et al. reported that the preoperative PLR was a good predictor for complications in gastric cancer patients after curative gastrectomy.

Although many studies have revealed the relationship of NLR with clinical outcomes in NSCLC, most of them only looked at the preoperative index and did not explore the association of postoperative dynamic changes in the ratio with postoperative outcomes. Our analysis of the trajectory of systemic inflammation scores such as NLR revealed a significant reduction on the fifth postoperative day in the RATS group compared to the thoracotomy group. The significantly lower NLR observed on postoperative day 5 in the robotic group likely reflects the reduced surgical trauma and faster recovery associated with minimally invasive approaches. Robotic surgery, by minimizing chest wall manipulation and overall inflammatory response, may contribute to a more favorable immunologic profile in the early postoperative period. This difference supports the growing evidence that less invasive techniques can positively influence systemic inflammation and potentially enhance postoperative recovery. Furthermore, the V postop NLR (V-NLR) was an independent predictive factor of postoperative complications. The mechanisms by which changes in NLR predict postoperative complications have not been fully understood. NLR is a well-known for reflecting systemic inflammation and infection. We assume that elevated NLR is a very early laboratory signal for acute inflammatory responses and infections. The shorter hospital stays observed in the RATS group are consistent with the findings of Veronesi et al., who reported reduced hospitalization times in patients undergoing robotic surgery for locally advanced or oligometastatic NSCLC after neoadjuvant treatment (18). Shorter hospital stays are beneficial not only for patient recovery and satisfaction but also for reducing healthcare costs and resource utilization (19).

Despite these promising findings, our study has limitations that should be acknowledged. The monocentric and retrospective design may introduce selection bias and limit the generalizability of the results. The relatively small sample size further underscores the need for larger, multicentric studies to validate our findings and provide more robust evidence regarding the benefits of RATS in locally advanced NSCLC. A critical limitation is the imbalance in the use of preoperative radiotherapy, which was administered to over one-third of patients in the thoracotomy group but to none in the robotic group. This discrepancy likely influenced postoperative outcomes such as complications, inflammatory response, and drainage volumes. Although reflective of real-world clinical practice and current selection criteria, this radiotherapy-related selection bias must be considered when interpreting our results. Additionally, the heterogeneity in neoadjuvant treatments (chemotherapy, chemo-immunotherapy, and targeted therapy) may also affect outcomes. Future studies should aim to stratify results by treatment regimen and control for preoperative radiotherapy more rigorously.

In conclusion, our study provides evidence that robotic surgery offers significant advantages over traditional open thoracotomy in managing locally advanced resectable NSCLC following neoadjuvant treatment. The observed benefits include fewer postoperative complications, shorter hospital stays, reduced daily drainage volumes, and a more favorable systemic inflammatory response as indicated by NLR.

However, the absence of preoperative radiotherapy in the robotic group represents a selection bias that may have contributed to these findings. This limitation must be acknowledged when interpreting the potential superiority of the robotic approach. Despite this, our results support the safety and feasibility of RATS for this patient population and highlight its potential for improved outcomes. Future prospective studies with larger, balanced cohorts are needed to confirm these results and assess the long-term benefits of RATS in NSCLC management.

## Data Availability

The raw data supporting the conclusions of this article will be made available by the authors, without undue reservation.
